# Mitochondrial Dysfunction Accelerates Ageing

**DOI:** 10.20900/immunometab20200035

**Published:** 2020-10-16

**Authors:** Johannes Schroth, Sian M. Henson

**Affiliations:** Translational Medicine and Therapeutics, William Harvey Research Institute, Barts and The London School of Medicine and Dentistry, Queen Mary University of London, Charterhouse Square, London EC1M 6BQ, UK

**Keywords:** ageing, mitochondria, metabolism, T cell, senescence

## Abstract

We review here the seminal findings of Desdin-Mico et al. showing that T cells with dysfunctional mitochondria induce multimorbity and premature senescence, due to mitochondrial transcription factor A (TFAM). They add further weight to the idea that targeting immunometabolism could be beneficial in combating the detrimental effects of age-related disease.

Mitochondrial dysfunction is a key event in many pathologies and contributes to the ageing process. Mitochondria have been shown to participate in every aspect of ageing, from a decline in stem cell function and cellular senescence, through to the development of the low grade inflammatory state [1]. Alterations that occur to mitochondria with age are numerous and can be observed in many different cells and tissues [2,3]. Indeed, we have shown that human CD8+ T cells were more susceptible to senescence compared to their CD4+ counterparts as they displayed a lower mitochondrial content and postulated loss of mitochondrial function controls the senescence phenotype in T cells [4] as well as other cell types [5,6]. However the mechanism remained elusive, that is until the recent paper by Desdin-Mico et al. published in Science demonstrated that mitochondrial dysfunction was controlled by mitochondrial transcription factor A (TFAM) [7].

In order to examine the links between mitochondrial loss and ageing, Desdin-Mico et al. used TFAM deficient mice, Tfamfl/flCd4Cre. TFAM is a nuclear gene that controls the stabilisation and replication of mitochondrial DNA. They found T cell mitochondrial content declined along with the loss of components of the electron transport chain causing a switch in T cell metabolism towards glycolysis. Interestingly they found young Tfamfl/flCd4Cre mice had a metabolic profile that resembled wild type 22 month mice, which was associated with Th1 skewing and increased expression of the Th1 master regulator T-bet. Additionally Tfamfl/flCd4Cre mice were also immunocompromised succumbing to acute infection with highly virulent mouse poxvirus, while young wild type animals survived infection both Tfamfl/flCd4Cre and old mice failed to resolve the infection.

Further evidence for TFAM being associated with ageing came from the observation that 7 month Tfamfl/flCd4Cre mice had an elevated inflammatory burden or inflammageing more usually seen in older animals. This increased inflammation is a predictor of multimorbidity during ageing and Tfamfl/flCd4Cre mice were found to have premature loss of muscular, cardiovascular and cognitive fitness together with a shorten life span. TFAM deficient animals were found to be less active and slower with less hypodermal fat than controls despite a higher energy expenditure.

The authors validated that this multimorbidity phenotype was due to a mitochondrial defect specifically in T cells by creating a T cell-specific Tfam deficient mouse model, Tfamfl/flLckCre. These animals also showed a prematurely aged phenotype by elevated expression of the senescence-associated markers p21 and p53. Incubation of hepatocytes or preadipocytes with serum from Tfamfl/flCd4Cre animals or with TNFα also increased p21 expression, supporting the idea that inflammation induces senescence and premature ageing. They also gave Tfamfl/flCd4Cre animals nicotinamide riboside (NR), the NAD+ precursor that declines with age and is a metabolic cofactor with a critical role in mitochondrial function. The use of NR was found to be protective against premature senescence and most but not all features of multimorbidity (Figure 1).

The paper concludes that T cells are capable of regulating both health and lifespan as well as highlighting the importance of tight immunometabolic control during ageing and the onset of age-related diseases. Finally, this work cements the idea that mitochondria play a causal role in senescence and that increasing mitochondrial biogenesis when coupled with mitochondrial degradation confers a survival advantage at both the cellular and organismal level.

## Figures and Tables

**Figure 1:**
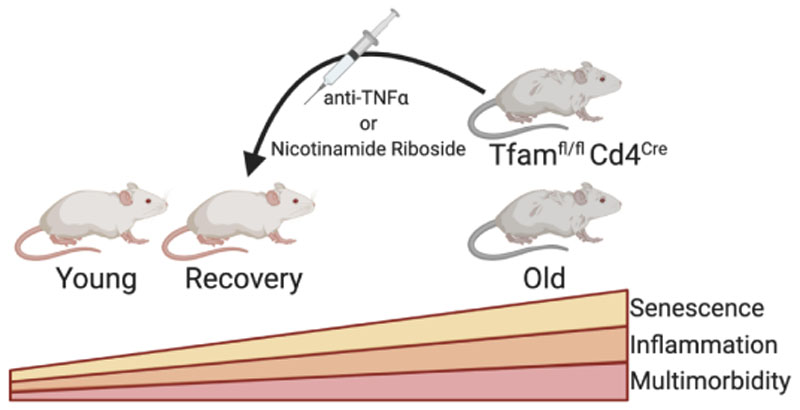
Summary of the work performed by Desdin-Mico et al. where they found mitochondrial dysfunction in T cells induced premature senescence and multimorbity due to a T cell specific deletion of mitochondrial transcription factor A (TFAM).
